# Preferences of UK-Based Dentists When Undertaking Root Coverage and Regenerative Procedures: A Pilot Questionnaire Study

**DOI:** 10.1155/2014/548519

**Published:** 2014-01-28

**Authors:** M. Siaili, D. Chatzopoulou, D. G. Gillam

**Affiliations:** Institute of Dentistry, Centre for Adult Oral Health, Barts and the London School of Medicine and Dentistry, Queen Mary University of London, London E1 2AD, UK

## Abstract

*Objectives*. The purpose of the present study was to evaluate the awareness and preferences of registered United Kingdom (UK) dentists specialising or with an interest in periodontics in root coverage and regenerative procedures. *Methods*. A cross-sectional postal survey of 366 dentists was conducted. The questionnaire was divided in two sections and most of the questions were giving the option of multiple answers. Frequencies and associations between the demographic profiles of the participants with their answers were evaluated. *Results*. 63% of dentists with an interest in periodontics and 32% of specialists returned the questionnaire. Guided tissue regeneration procedures and the use of enamel matrix derivatives were recommended for the reconstruction of bony defects and both subepithelial connective tissue graft and coronally advanced flap with or without enamel matrix derivatives were the most popular choices for root coverage. Smoking was considered a contraindication by most of the participants and conflicting responses were given regarding the use of antibiotics as part of the care following regenerative procedures. *Conclusions*. The participants incorporated both traditional and “novel” techniques and products in reconstructive procedures and appeared to be up to date with the evidence from the dental literature. However, it was evident that there was confusion regarding the role of antibiotics in regenerative procedures.

## 1. Introduction

Reconstructive periodontal surgery has been one of the most dynamic therapeutic procedures in periodontology over the last three decades, and yet, the ultimate goal of regeneration of the periodontal supporting tissues remains unpredictable and challenging. Previous surveys have reported on the regeneration of intrabony defects with enamel matrix derivatives (EMD) [1] and on the coverage of denuded roots using a variety of surgical techniques and products [2].

Guided tissue regeneration (GTR) procedures with or without bone grafting materials would appear to be the recognised gold standard for the reconstruction of intrabony and interradicular defects for more than one decade [3]. The emergence of tissue engineering science has also enabled the development of various biomaterials for clinical use; for example, EMD have been reported to be more effective than other biomaterials and have been shown to have similar efficacy with GTR techniques [4]. Combining different materials may also be of benefit and some studies have demonstrated encouraging results [5]. According to Cortellini and Tonetti (2009) [6], both the flap design and flap management are important when assessing outcomes of osseous reconstructive techniques and as a consequence various flap designs have been developed with a view to using a minimally invasive approach.

Although the subepithelial connective tissue graft (SCTG) has demonstrated outcomes of complete root coverage, it is not the only efficacious available method [7]. For example, the coronally advanced flap (CAF) with or without EMD [7] has been demonstrated to cover the root surface in addition to other procedures such as the free gingival graft (FGG) as part of a two-stage approach [8].

A number of factors, concerning the patient and the surgical site have to be evaluated prior to the application of any regenerative procedure and subsequently be controlled during the postoperative healing period [9, 10]. There are however limited data regarding the acceptance of the research outcomes from these procedures in mainstream clinical practice. Therefore, the purpose of the present study was to evaluate whether UK dentists with a special interest or specialty in periodontics were conversant with current research outcomes in periodontal regeneration techniques and procedures.

## 2. Materials and Methods

### 2.1. Study Design and Participants

A validation stage using a cross-sectional self-administered questionnaire preceded the pilot survey, for which 80 questionnaires were given to dentists of different clinical disciplines with an interest in periodontics. The participants were either staff members or postgraduate students in the Centre for Adult Oral Health at the Bart's and The London School of Medicine and Dentistry, QMUL, London, UK. A reminder was provided to all the participants after one month and a further period of two months was allowed for the return of the questionnaires. Initial analysis of the pilot data enabled the investigators to improve only the final layout of the questionnaire prior to sending it out to the practising dentists but not the questions *per se.*

The final version of the questionnaire was conducted using a sample of 286 dentists, registered as specialists in periodontics on the list of the General Dental Council of United Kingdom. A four-month period was allowed for responses and no subsequent reminders were sent. All questionnaires returned within the four month period were included in the analysis.

### 2.2. Questionnaire

The questionnaire was divided in two sections. Most of the questions had a multiple-choice format and some were open-ended or dichotomous in nature.

The first section was designed to collect information regarding the profile of the dentists and consisted of six questions. The questions addressed the dentists' age, gender, specialty (periodontics, general dentistry, implantology, or other) and the year of graduation. The number of subscriptions to periodontal journals, the interest of the dentists in performing periodontal regenerative procedures on a numerical scale from 1 (no interest) to 10 (high interest), and an estimate of the regenerative procedures performed annually were also requested.

The second section (15 questions) dealt with general questions on periodontal regeneration, the site-specific factors taken in consideration during the pre- and postsurgical assessment of the treated area together with the types of regenerative materials used by dentists. Further questions regarding the surgical management of marginal tissue recession defects with relevant clinical photographs (in colour) and simplified diagrams of four selected clinical case scenarios with labial marginal tissue recession defects of different stages (Miller class I–IV) were asked in this section of the questionnaire. A choice of clinical procedures was provided for each of the questions: (1) CAF with or without EMD, (2) SCTG, (3) FGG, (4) laterally positioned flap (LPF), (5) double papilla flap (DPF), (6) GTR, and/or (7) other treatment. A further four clinical photographs (in colour) and simplified diagrams of three-, two-, one-wall intrabony defects and class II furcation defects required the dentists to provide a response on the clinical management of the clinical scenario. Various treatment choices were provided for each of the clinical scenarios, for example, (1) open flap debridement only (OFD), (2) resective surgery, (3) GTR, (4) bone graft with or without barrier membrane, (5) EMD with or without bone fillers, and/or (6) other option. Furthermore, questions regarding the frequency of use of EMD on a monthly basis and the utilization of special flap designs during periodontal regeneration procedures were also required. Questions relating to the exclusion of smokers from regenerative procedures and whether systemic antimicrobials should be prescribed as part of the postoperative care were also requested. The participants were finally asked for an estimate of their patients' acceptance of using animal derived regenerative materials.

### 2.3. Statistical Analysis

Data were analysed using the SPSS Version 18 software (IBM, Portsmouth, UK) and frequency tables were constructed. For the description of the data, the median, standard deviations, minimum, and maximum values regarding the continuous data and frequencies and relative frequencies (proportions) for categorical data were calculated.

Several hypothetical clinical scenarios were formulated in order to evaluate the association between the selected characteristics of the participants' profile and their attitudes and preferences when undertaking regenerative procedures (if relevant). Data that differed significantly from a normal distribution as assessed using the Kolmogorov-Smirnov test were subsequently assessed using nonparametric tests for any associations between the variables.

The One-Way ANOVA test was used to evaluate the relationship between numerical and categorical variables. The Chi-square test was applied for the assessment of the association of two categorical variables.

## 3. Results

A total of 91 specialists out of 286 (31.9%) and 50 out of 80 (62.5%) dentists with a special interest in periodontics returned their questionnaires and 141 returned questionnaires were included in the final statistical analysis. The participants' profiles are summarized in Table 1.

Most of the participants reported paying a subscription to one periodontal journal (37%; *n* = 52), whereas 20% (*n* = 28) subscribed to two and 11% (*n* = 16) to three periodontal journals. The mean interest in performing regenerative procedures was high (7.57 ± 0.2). There was a relationship between “years since graduation” and “interest in regenerative procedures” with the exception of the groups that had experience of over than 30 years since graduation. A statistically significant relationship between the number of subscriptions to periodontal journals and the interest of the participants in periodontal regenerative procedures was demonstrated (*P* < 0.001). With the increase in the rate of subscription to periodontal journals, an increase in the rate of interest in regenerative procedures was also noted.

**Figure 1:**
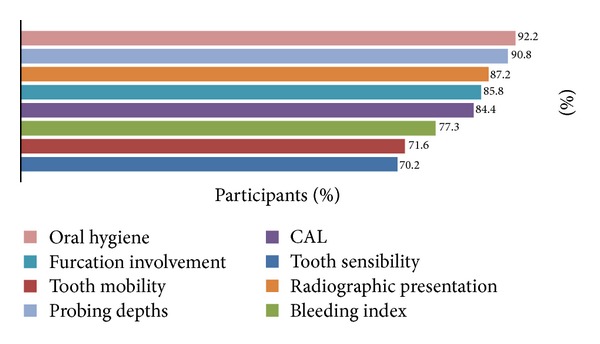
Participants' responses: frequencies of the parameters routinely evaluated prior to and following periodontal regeneration procedures.

The mean percentage of periodontal regenerative procedures from the overall total of treated cases was 14% (±1.96).  Figure 1 provides information regarding the parameters evaluated prior to and following a periodontal regenerative procedure. Most of the participants (92%; *n* = 130) indicated that oral hygiene was the main routine factor for evaluation. Regarding the techniques and materials utilised for the regeneration of intrabony defects, the EMD material was the predominant choice (65%; *n* = 91) and 54% (*n* = 76) of the participants indicated that they would use GTR with an absorbable barrier membrane.

**Figure 2:**
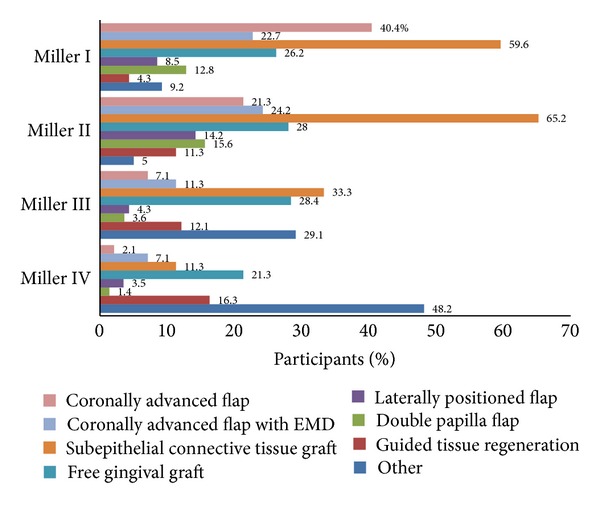
Participants' responses: frequencies of the options for the treatment of Miller Class I–IV marginal tissue recession defects.

The chosen treatment modalities for the four selected clinical situations presented relative to the Miller I–IV marginal tissue recessions are summarised in Figure 2. For the selected clinical scenarios of Miller class I and II marginal tissue recession defects, the use of SCTG was the most favourite treatment option suggested by the participants (60%; *n* = 84 and 65%; *n* = 92, resp.).

Responses to the selected clinical scenario of a Miller class III recession defect indicated the frequent use of SCTG (33%; *n* = 47) and FGG (23%; *n* = 40), the majority of participants (48%; *n* = 68) also considered the other available treatment options as preferable, for example, “nonsurgical treatment” (36%; *n* = 51), “extraction” (7%; *n* = 10), “monitoring” (1%; *n* = 2), “single tufted toothbrush” (1%; *n* = 2), and use of “platelet-derived growth factor (PDGF) and bone substitute(s)” (0.7%; *n* = 1).

**Figure 3:**
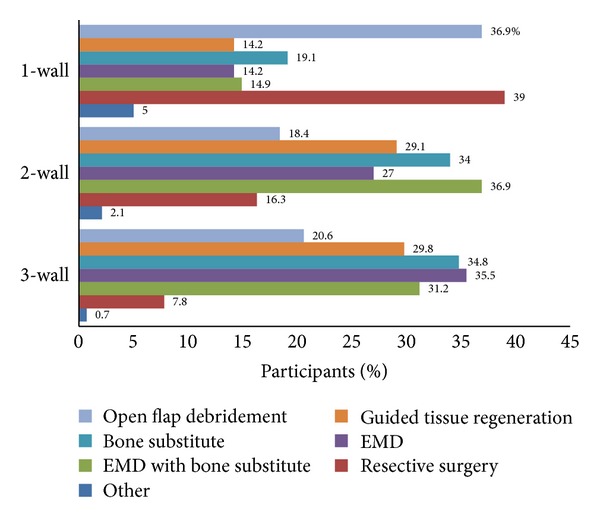
Participants' responses: frequencies of options for the treatment of three-, two-, and one-wall intrabony defects.

The preferences of the participants for the surgical treatment of intrabony defects are shown in Figure 3. For the correction of a three-wall intrabony defect, most of the participants opted for using EMD without (36%; *n* = 50) and with bone grafts (31%; *n* = 44) and using bone grafts with or without the use of barrier membranes (35%; *n* = 49).

The four main responses for the selected clinical example of a 2-wall intrabony defect were (1) EMD combined with bone grafts (37%; *n* = 52), (2) bone grafts with or without barrier membranes (34%; *n* = 48), (3) GTR with resorbable membranes (29%; *n* = 41), and (4) EMD alone (27%; *n* = 38).

For the reconstruction of the one-wall intrabony defect, the resective surgery (39%; *n* = 55) and the OFD (37%; *n* = 52) options were proposed.

The responses to the selected clinical case resembling a Class II furcation defect indicated that the majority of participants would favour the use of (1) EMD alone (33%; *n* = 47), followed by (2) GTR with the use of resorbable barrier membranes (31%; *n* = 43), (3) OFD alone (24%; *n* = 34), (4) EMD and bone grafts (23% *n* = 33), (5) resective surgery (23%; *n* = 32), and (6) bone grafts with or without barrier membranes (19%; *n* = 27).

Most of the participants reported that they would use EMD products for the regeneration of periodontal defects “one to three times” per month (48%; *n* = 67).

More than 70% of the participants indicated that they would use a special flap design in regenerative procedures for bony defects. The papilla preservation flap and the coronally advanced flaps were reported more frequently than the minimally invasive surgical technique. In addition, it was observed that with the increase of the number of subscriptions to periodontal journals, the frequency of utilisation of special flap designs was also increasing (*P* < 0.001).

Smoking was, however, considered a contraindication for regenerative procedures by 65% (*n* = 91) of the participants due to the various features reported in the published literature, for example, vasoconstriction, impaired postoperative healing, and compromised outcomes.

Approximately 35% (*n* = 49) of the participants stated that they would not prescribe antibiotics as part of a patient's postoperative care following regenerative procedures. 21% (*n* = 30) of the participants however indicated that they would use antibiotics for more than 90% of their treated cases.

**Figure 4:**
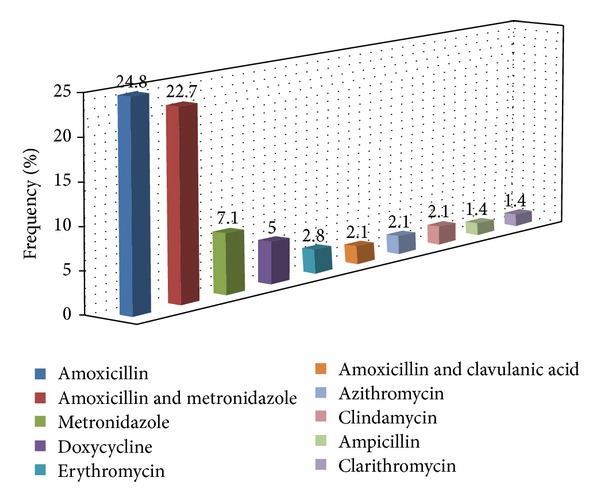
Participants' responses: The use of systemic antibiotics as part of the postoperative care following regenerative procedures.

The two predominant responses to the question regarding the type of antibiotics prescribed postoperatively were amoxicillin alone (25%; *n* = 35) and the combination of amoxicillin and metronidazole (23%; *n* = 32) (Figure 4).

The patients' rejection of animal derived materials was infrequent as reported by the majority of the participants (38%; *n* = 53) indicating that their patients had never rejected treatment with an animal-derived material. 18% (*n* = 25) of the participants indicated that <5% of their patients would reject the material.

## 4. Discussion

The response from the validation phase was higher than from the pilot study. This may be attributed to a number of factors, for example, the close relationship between the investigators and the staff members in the hospital and the reminder given to all the participants of the pilot study after one month. Within the limitations of this study and the main issue of limited funding and time, a reminder was not sent to the specialist practices at the stage of the pilot study. However, a future main study should incorporate reminding the participants either by the postal, electronic mail, or telephone route, and alternatively a web based questionnaire could also be utilised.

The majority of the participants appeared to share a common knowledge and clinical awareness regarding the factors that may be considered prior to and following a regenerative procedure. It was observed that almost all participants would assess the ability of a patient in maintaining a high level of oral hygiene which is in agreement with previously reported survey studies as well as the substantive published evidence about periodontal health [1, 11, 12]. One of the other factors which were frequently chosen by the participants was the radiographic presentation of the treated site. However, one would expect clinical attachment measurements to have featured more prominently in the participants' responses since a precise and complete clinical examination should always precede any radiographic assessment of a site [13].

The participants' attitudes concerning root coverage procedures may be demonstrated by presenting the results in two groups, namely, (1) the choices regarding the defect types with the potential of “predictable” outcomes (Miller class I and II recession defects) and (2) the choices referring to defect types with the “least predictable” treatment outcomes (Miller class III and IV). For the first group SCTG and CAF with or without EMD were the most prominent choices. Only in Miller class II defects the FGG procedure was frequently selected in addition to the three aforementioned techniques. These results are in accordance with the evidence from the published literature that demonstrate the superiority of CAF with or without EMD and the SCTG in root coverage procedures [7, 9, 14]. The results from the second group are also consistent with the unpredictable treatment outcomes of more advanced defects [15]. The response rates on the use of the so-called “gold standard” SCTG remained high when treating Miller class III and reduced dramatically when treating Miller class IV marginal tissue recession defects. The use of FGG followed by GTR was also considered to be the dominant option regarding the treatment of the “least predictable” defects. Alternative suggestions on “no surgical treatment,” “monitoring,” or “extraction” of the affected teeth were also frequently made by the participants. The loss of the interdental tissues in the second group of defects was correctly considered by the participants as a factor that could compromise the results of any attempt for root coverage [16]. The present study also demonstrated an overall poor acceptance of GTR techniques in the management of root coverage procedures, a similar finding with the Zaher et al. survey [2]. There was also a low preference for DPF and LPF techniques although these techniques were considered as effective in some studies [17].

For the management of intrabony defects, a strong tendency for considering the number of residual bony walls as the determinant factor for the choice of the regenerative method (filling materials or not) was reported [18, 19]. The participants reported that they would use different treatment protocols for the management of self-contained defects (three-wall) in comparison to defects of “less favourable” anatomy (two-wall, one-wall) with an orientation to regeneration and resection, respectively. However, this observation shows no consideration of recent studies which have demonstrated that GTR procedures can be effective in the reconstruction of one-wall (and wide) defects [20, 21]. Evidence from the published literature appears to demonstrate that both demineralised freeze dried bone allografts (DFDBA) and autogenous grafts support the formation of a new attachment apparatus in intrabony defects, whereas alloplastic graft materials support the formation of repair than regeneration [22, 23]. However, the preferred choice in this study appeared to be the use of xenografts, which may be due to the popularity of commercially available products of bovine origin with established long-term efficacy [24] and the nonapproved use of DFDBA products in the UK. The indicated use of EMD “one to three” times on a monthly basis, as observed in the present study, was consistent with the results from the Schröen et al. study [1]. However, the proposed hypothesis that younger participants would prefer to use EMD more often that their more experienced colleagues was not substantiated from the present study. Overall, this study highlights the lack of consensus for the biomaterial of choice when treating intraosseous defects [25].

According to the results from published studies, the flap design is of critical importance in regenerative procedures as it should facilitate both full coverage and wound stability during the healing process [6]. The majority of the participants in this study advocated the use of special flap designs in regenerative procedures. The well established techniques of papilla preservation flaps (PPF) and CAF were the most frequent suggestions, whereas the more recently introduced minimally invasive surgical technique (MIST) was infrequently mentioned [26, 27]. Interestingly, an association between the frequency of the use of special flap techniques and the subscription to periodontal journals was also observed and one could speculate that this would suggest participants with more subscriptions to journals were more up-to-date regarding current surgical protocols and techniques which may as a consequence improve their clinical practice. Currently, regenerative procedures appear to be minimally invasive in nature and as such technically more demanding for the clinician. In retrospect, it would have been of interest to see whether the participants would include the use of specialised equipment, for example, loupes, operating microscope, or microsurgical instruments in association with special flap designs [28].

The majority of the participants in the present study appeared to be aware of the negative effects of smoking on the successful outcome of regenerative procedures [29, 30]. The heightened awareness of the participants regarding the significance of smoking in “healing inhibition” and hence in “compromised treatment outcomes” was also evident in the present study [31]. However, no conclusive evidence exists in the published dental literature regarding the odds ratio which describes the strength of association between the effect of smoking and the outcomes of regenerative procedures together with the dose related effect of smoking in regenerative procedures. Hence, the questionnaire used in the present study did not address the importance of the number of smoked cigarettes per day and the total years of smoking in contrast to the Schröen et al. study [1].

Although several published studies reported on the benefits of the adjunctive administration of systemic antibiotics [32], other investigators however have failed to demonstrate any additional, consistent, and significant benefits in terms of clinical parameters [33, 34]. From the present study, it was evident that there was some confusion regarding the clinical efficacy of systemic antimicrobials in periodontal reconstructive therapy. The criteria according to which the dentists may use or not use antibiotics following regenerative procedures should be addressed in a future study. Concerning the prescribed antibiotic type, it appeared that participants based their choice on empirical use rather than the evidence-based knowledge. Although the antibiotic of choice was amoxicillin which is in agreement with some studies [35], it was difficult to discern a clear pattern of the prescribed use of the antimicrobials.

The rejection rate of animal derived materials was low. The results from the present study would therefore appear to be in agreement with the Swiss study reported above [1]. One could therefore speculate that the rejection/acceptance of animal derived materials between various countries and regions in a country may be dependent on the particular cultural, religious, and general profile of its citizens.

The use of antibiotics, however, needs to be clarified and therefore future research may be directed towards the evaluation of the efficacy of the adjunctive use of systemic antibiotics in periodontal regenerative therapy. One possible outcome from these studies would be the recommendation of specific protocols and treatment regimes for using systemic antibiotics in regenerative procedures. Additionally, future questionnaire-based surveys could compare attitudes and trends regarding periodontal treatment in different countries and with larger samples. A particularly challenging and yet valuable aspect of a questionnaire-based study would be to include questions in terms of outcome measures such as success rate, postoperative morbidity, and the cost effectiveness of a periodontal (regenerative) regime.

## 5. Conclusions

UK-based dentists with either a special interest or a specialty in periodontics incorporated both traditional and “novel” techniques and products when undertaking reconstructive procedures and appeared to be up to date with the evidence from the available dental literature. However, further research is required to understand the preferences of the specialists around different countries and to clarify the role of antibiotics in periodontal regeneration procedures as it was evident from the present study that there was confusion among dentists regarding this role.

## 6. Clinical Relevance

### 6.1. Scientific Rational for Study

A large number of surgical techniques and products are available for the surgical reconstruction of periodontal tissues. This study used a questionnaire to evaluate the views of practicing clinicians in UK regarding the regenerative procedures in a clinical environment.

### 6.2. Principal Findings

The dentists in UK with interest or with a speciality in periodontology show a high interest in the reconstruction of the lost periodontal tissues and use a variety of procedures to achieve this.

### 6.3. Clinical Implications

A number of factors other than the proven in the dental literature efficacy may influence the use of a treatment modality in the every-day clinical practise. For example, the financial cost of a procedure may influence the clinician's choice.

## Figures and Tables

**Table 1:** General characteristics of the participants.

	Relative frequency (%), frequency (*n*)
Number of responders:	
Hospital staff	47.1 (64)
Private practice	52.9 (72)
Gender:	
Male	62.2 (84)
Female	37.8 (51)
Age (years)	Mean: 44 ± 1.05; 26–74
20–29	14.8 (20)
30–39	23.0 (31)
40–49	26.7 (36)
50–59	23.7 (32)
60–65	6.7 (9)
65	5.2 (7)
Years since graduation	Mean: 20 ± 1.04; 2–50
5	14.2 (20)
6–9	12.1 (17)
10–19	20.6 (29)
20–29	25.5 (36)
30–39	15.6 (22)
>40	7.8 (11)
